# Supplementary material for: Use of Microalgae-Derived Astaxanthin to Improve Cytoprotective Capacity in the Ileum of Heat-Induced Oxidative Stressed Broilers
Source: Animals (Basel). 2024 Jun 29;14(13):1932. doi: 10.3390/ani14131932 (PMC11240551; doi:10.3390/ani14131932)
Supplement: Supplementary file 1 [file animals-14-01932-s001.zip › animals-3056691-supplementary.pdf]

Table S1. Common components of *Haematococcus* algae (Lorenz; Cyanotech 1999).

	Minimum	Maximum	Mean
Protein	17.30	27.16	23.62
Carbohydrates	36.9	40.0	38.0
Fat	7.14	21.22	13.80
Iron (%)	0.14	1.0	0.73
Moisture	3.0	9.0	6.0
Magnesium (%)	0.85	1.4	1.14
Calcium (%)	0.93	3.3	1.58
Biotin (mg/lb)	0.108	0.665	0.337
L-carnitine (ug/g)	7.0	12	7.5
Folic acid (mg/100g)	0.936	1.48	1.30
Niacin (mg/lb)	20.2	35.2	29.8
Pantothenic acid (mg/lb)	2.80	10.57	6.14
Vitamin B1 (mg/lb)	<0.050	4.81	2.17
Vitamin B2 (mg/lb)	5.17	9.36	7.67
Vitamin B6 (mg/lb)	0.659	4.5	1.63
Vitamin B12 (mg/lb)	0.381	0.912	0.549
Vitamin C (mg/lb)	6.42	82.7	38.86
Vitamin E (IU/lb)	58.4	333	186.1
Ash	11.07	24.47	17.71
